# Construct validity and reliability of the 2-minute step test in patients with knee osteoarthritis

**DOI:** 10.1186/s12891-022-05114-1

**Published:** 2022-02-17

**Authors:** Thiago Felipe de Morais Almeida, Almir Vieira Dibai-Filho, Fernanda de Freitas Thomaz, Eloiza Adelaide Amaral Lima, Christian Emmanuel Torres Cabido

**Affiliations:** 1 Postgraduate Program in Physical Education, Universidade Federal do Maranhão, Núcleo de Esportes, Primeiro andar, Avenida dos Portugueses, 1966, Vila Bacanga, São Luís, MA 65080805 Brazil; 2 Hospital Sarah, São Luís, MA Brazil; 3 Instituto de Coloproctologia, São Luís, MA Brazil

**Keywords:** Reproducibility of results, Chronic pain, Exercise test

## Abstract

**Objective:**

To validate and evaluate the intra- and inter-rater reliability of the 2-min step test (2MST) in measuring the functional performance of patients with knee pain associated with osteoarthritis (OA).

**Methods:**

Forty-one patients with knee OA was included. Two examiners assessed the patients at two times with interval between the test and retest from 7 to 14 days. All executions of 2MST were recorded in real time by the examiners and were also recorded by video. The intraclass correlation coefficient (ICC) and 95% confidence interval (CI), standard error of measurement (SEM) and minimum detectable difference (MDD) were used to determine reliability. In the construct validity, we correlate the score of the 2MST with the other instruments used in the study: The Western Ontario and McMaster Universities Osteoarthritis Index (WOMAC), Numerical Pain Scale (NPS), Pain-Related Catastrophizing Thoughts Scale (PCTS) and Chronic Pain Self-Efficacy Scale (PSEQ). The agreement between the face-to-face assessment and the evaluation based on the video record was assessed using the Bland-Altman methodology in the 4 moments of the 2MST.

**Results:**

2MST presented excellent intra- (ICC = 0.94, SEM = 4.47, MDD = 12.40) and inter-rater reliability (ICC = 0.97, SEM = 3.07, MDD = 8.52). The agreement was acceptable between face-to-face assessments and the analyzes performed on video. All instruments showed a statistically significant correlation with 2MST, except the PCTS. A correlation magnitude above 0.50 was found between the 2MST and pain and function domains of the WOMAC, and a correlation magnitude between 0.30 and 0.50 with the joint stiffness domain of the WOMAC, NRPS and PSEQ.

**Conclusion:**

2MST proved to be valid for assessing functional capacity in patients with knee OA, with excellent reliability.

## Introduction

Osteoarthritis (OA) is a chronic-degenerative joint disease that is evidenced by the wear of the joint cartilage [1, 2]. In the clinical evaluation of patients with knee pain associated with the presence of OA, subjective methods are usually used, such as The Western Ontario and McMaster Universities Osteoarthritis (WOMAC) and the Lequesne index, which are specific for this population [3]. The WOMAC is the most used questionnaire worldwide, considered the most appropriate to assess the functional capacities and limitations related to physical aspects [4]. Additionally, psychosocial aspects need to be considered in the clinical assessment of patients [5, 6].

Associated with subjective methods, objective tests are used to assess functional capacity, commonly in assessment routines in rehabilitation centers and in research. Six-minute walk tests (6MWT), Timed Up and Go (TUG), gait speed tests, Chair-Stand Test (CST), and Stair-Climb Test are recommended for patients with knee OA [7, 8], although there is no test considered the gold standard for assessing functional capacity in patients with knee OA.

In addition to these, another test that measures functional capacity and is applicable in some populations, such as elderly people of both sexes [9, 10], hypertensive elderly and elderly people with mild cognitive impairment, is the two-minute step test (2MST) [9, 11–14]. Previous studies have found similar results between 2MST and 6MWT [10] and a weak to moderate correlation of 2MST with 6MWT and TUG in hypertensive older adults [14].

When compared to the TUG and 6MWT tests, the 2MST presents the positive aspects of being of low cost, quick execution and not requiring large spaces or specific furniture for its realization. 2MST only needs a wall for its execution, which within the routine of a rehabilitation service facilitates the evaluative dynamics, since its execution can be performed inside the outpatient clinic, inpatient room or in the corridor. However, there is still a lack in the literature on construct validity and reliability of the use of 2MST in patients with knee OA, despite being a promising tool to investigate functional capacity.

The clinical plausibility for the application of 2MST in patients with knee OA is related to the importance of the climbing up and down movements, as explained below: the movement during the 2MST performance involves the knee and hip joints; stair climbing is one of the first task affected in patients with knee OA [15]; and lower stair-climbing speed is commonly seen in patients with knee OA [16, 17].

Given this scenario, the present study aimed to assess the construct validity and intra- and inter-examiner reliability of the 2MST in measuring the functional performance of patients with knee pain associated with OA. The hypothesis of this study is that 2MST is a reliable and valid measure for the population tested.

## Methods

### Study design

This is a study of the construct validity and reliability of the 2MST. The research was carried out in the Adult Orthopedic Rehabilitation sector of Hospital Sarah (São Luís, MA, Brazil) from July 1, 2020 to January 30, 2021, approved by the institution’s research ethics committee (protocol number 3.962.645). All research participants validated their participation by signing an informed consent form.

### Participants

The sample calculation considered the confidence coefficient of 0.95 and the amplitude of the confidence interval for the intraclass correlation coefficient (ICC) of 0.30. In addition, the calculation was performed to detect adequate reliability (ICC = 0.75) according to the classification of Fleiss [18]. Thus, a sample size of 34 participants was estimated. To compensate for a possible sample loss, a minimum sample size of 40 volunteers was considered. The processing of the sample calculation was carried out based on the study carried out by Bonnett [19].

We included in this study: patients of both genders; with a minimum age of 40 years and a maximum of 80 years; complaint of knee pain lasting more than 3 months, diagnosis of knee OA issued after evaluation by an experienced orthopedist, based on criteria established by the American College of Rheumatology with clinical evaluation and imaging. The criteria were presence of pain, presence of osteophytes and at least one of the 3 characteristics (age over 50 years, presence of crackling and/or morning stiffness for less than 30 min). Patients with grade 2 or 3 in the classification of Kellgren and Lawrence were included in the study [20].

The non-inclusion criteria adopted in the study were: individuals with a history of lower limb surgery; use of mobility aids; neurological disorder (sensory and/or motor); hip OA; use of prosthesis or orthosis in the lower limbs; cardiopulmonary diseases or any other acute adverse health condition that may make it impossible to carry out the proposed tests. Exclusion criteria were patients who did not show up within the stipulated period of 7 to 14 days for the retest.

### Assessment procedures

The present study was integrated by two physical therapist examiners who performed the measurements with the 2MST independently in two moments (test and retest), resulting in a total of 4 test applications for each participant, two evaluations on the first day and two more on the second day. The assessments were carried out by two physiotherapists with more than 10 years of experience. In addition, a 1-month prior training was carried out to standardize the execution of the tests.

When measuring functional capacity using 2MST, the examiner measured the maximum number of knee lifts that the individual performs in 2 min. Before starting the test, a marking was made on the wall, at the midpoint between the patella and the anterosuperior iliac spine. The examiner counted the number of right knee elevations that reached this mark for patients who had pain associated with right knee OA and for patients with bilateral symptomatic knee OA. The counting of left knee elevations was performed only in patients with exclusive symptoms of left OA.

Two previous runs of the test were performed for familiarization, for a period of 30 s (with a 1-min rest interval between them). After 1 min of rest, the first examiner (staying beside the patient for safety in case of imbalance) applied the test for 2 min, giving verbal information to start the test, another when 1 min had passed and when there were 30 s to the end of the test. After a 10-min rest break, the second examiner performed the same procedure. The order of examiners was defined by drawing lots before each application of 2MST.

After a minimum interval of 7 days and a maximum of 14 days, the patients were evaluated with the 2MST again by the two examiners. The same pattern performed in the test was maintained, with the maintenance of the time, in the same environment, without the patient having performed any type of physical exercise on the day of the assessment, in order to avoid fatigue before the assessment.

All 2MST runs were recorded for review by the examiners. In addition, the planes were filmed for further analysis using an iPhone 8 cell phone (Cupertino, CA, USA) and a universal telescope tripod set at the height of the marking made on the wall. A third independent examiner counted the number of steps using video recordings. This measure was taken to allow for the analysis of agreement, considering video-based counting as the reference measure.

To determine the construct validity through correlations, patients answered validated instruments, translated and adapted to Brazilian Portuguese, commonly used in patients diagnosed with knee OA. Therefore, we used several questionnaires and scales to better assess the pain of patients with knee AO, within a biopsychosocial model, considering pain intensity, physical function, joint stiffness, catastrophizing and self-efficacy.

The Western Ontario and McMaster Universities Osteoarthritis Index (WOMAC) is a self-administered questionnaire designed specifically for individuals with knee or hip OA. It was culturally validated and adapted to Brazilian Portuguese [21]. The questionnaire has three domains: pain, with 5 items; joint stiffness, with 2 items; and physical function, with 17 items. For each item, the patient has 5 response options (none, mild, moderate, strong, very strong). The pain domain score ranges from 0 to 20, the stiffness domain ranges from 0 to 8 and the physical function domain ranges from 0 to 68 points. The higher the value, the worse the symptoms.

The Numerical Pain Scale (NPS) is a scale consisting of a sequence from 0 to 10, where the value 0 represents “no pain” and the number 10 represents “the worst pain imaginable”. Thus, individuals graded their pain based on this parameter. This scale is validated for Portuguese [22]. Each patient answered the scale twice: once for pain intensity at rest and once for pain intensity during active knee movements.

The Pain-Related Catastrophizing Thoughts Scale (PCTS) was used to assess catastrophizing in relation to pain. It is composed of 9 items scaled on a Likert scale, ranging from 0 to 5 associated with the words “almost never” and “almost always”. The total score is the sum of the scores of the completed items, divided by the number of these items answered, with the minimum score being 0 and the maximum 5. Higher scores indicate a greater presence of catastrophic thoughts. The scale was adapted and validated for Brazilian Portuguese [5].

The Pain Self-Efficacy Questionnaire (PSEQ) was developed to investigate the degree of confidence that patients with chronic pain have about themselves to perform daily activities or functions. It consists of 10 items, with response options ranging from 0 to 6, 0 being “not at all confident” and 6 “completely confident”, totaling a score from 0 to 60. The higher the score, the greater is your self-efficacy. This instrument is validated for Brazilian Portuguese [6].

### Statistical analysis

To characterize the sample, quantitative data were described as mean and standard deviation (SD), and qualitative data as number and percentage. The intraclass correlation coefficient (ICC_2,3_) was used to determine intra- and inter-examiner reliability, with its respective 95% confidence interval (CI), standard error of measurement (SEM) and minimal detectable difference (MDD) [23]. To interpret the ICC value, the study by Fleiss [18] was used as a reference: for values below 0.40, reliability was considered low; between 0.40 and 0.75, moderate; between 0.75 and 0.90, high, and, finally, values greater than 0.90, reliability was considered excellent.

To determine the construct validity, the Shapiro-Wilk normality test was initially applied. Upon identification of non-normal distribution of data, Spearman’s correlation coefficient (rho) was used to verify the magnitude of correlation between 2MST and NPRS, WOMAC, PCTS and PSEQ. As a hypothesis for the magnitudes of correlation, we expect a correlation ≥0.50 between 2MST and the physical function domain of the WOMAC (similar constructs) and a correlation ranging from 0.30 to 0.50 with the pain and joint stiffness domains of the WOMAC, NPRS, PCTS and PSEQ (related but different constructs). It is expected that at least 75% of the hypotheses defined a priori are confirmed [24].

The agreement between the face-to-face evaluations of the 2MST and the evaluation performed based on the video recording was analyzed using the Bland-Altman methodology, considering 4 moments of the completion of the 2MST [25].

The software used for the analyzes was SPSS (version 17, Chicago, IL, USA) and a significance level of 5% was considered.

## Results

Forty-three patients diagnosed with knee OA were included in the study, with a sample loss of two individuals who did not attend within the recommended period for the retest. Thus, the final sample consisted of 41 volunteers.

Thus, according to Table 1, the sample in this study was composed mostly of female adults, with overweight and bilateral knee OA, with grade 3 OA severity in the Kellgren and Lawrence classification. In addition, the mean duration of chronic knee pain symptoms was 50 months.

**Table 1:** Sociodemographic characteristics of patients with knee osteoarthritis (*n* = 41)

Variable	Mean (standard deviation) or n (%)
Age (years)	56.48 (7.60)
Sex (women)	35 (85.4%)
Body mass index (kg/m^2^)	30.51 (3.96)
Knee osteoarthritis	
Bilateral	37 (90.2%)
Right	2 (4.9%)
Left	2 (4.9%)
Education	
Basic education	8 (19.5%)
Complete primary education	5 (12.2%)
Complete high school	20 (48.8%)
Complete higher education	5 (12.2%)
Posgraduate	3 (7.3%)
Kellgren-Lawrence classification	
Grade 2	12 (29.3%)
Grade 3	29 (70.7%)
Chronicity of pain (months)	50.04 (44.34)

Table 2 describes the mean values and standard deviation of the scores obtained through the questionnaires applied in the study. Table 3 describes the 2MST values according to the two examiners and the measurement performed based on the recorded video of the test execution.

**Table 2:** Scores of the instruments applied in the study (*n* = 41)

Questionnaires	Mean (standard deviation)
Western Ontario and McMaster Universities Osteoarthritis Index	
Pain domain	11.21 (3.65)
Joint stiffness domain	4.63 (2.25)
Physical function domain	37.31 (11.54)
Numerical Pain Scale	
Rest	5.56 (2.88)
Movement	8.12 (2.22)
Pain-Related Catastrophizing Thoughts Scale	1.79 (1.37)
Pain Self-Efficacy Questionnaire	48.04 (11.81)

**Table 3:** Mean values and standard deviation of the execution of the 2-min step test (2MST) according to the examiners and in the video analysis (*n* = 41)

2MST	Examiner 1		Examiner 2	
	Test	Retest	Test	Retest
Face-to-face measurement	64.75 (19.03)	67.12 (18.27)	65.56 (18.70)	67.58 (19.11)
Measurement with video	64.73 (18.97)	67.39 (18.12)	65.46 (18.65)	67.68 (19.11)

Regarding reliability, we observed excellent ICC values (≥ 0.94) when considering different times and different examiners, as shown in Table 4. In turn, when considering the validity of 2MST, we observed a magnitude of correlation above 0.50 between 2MST and the WOMAC pain and function domains, and magnitude of correlation between 0.30 and 0.50 with the WOMAC joint stiffness domain, NPRS at rest and during movement, and PSEQ (Table 5).

**Table 4:** Intra- and inter-examiner reliability of the 2-min step test (2MST) (*n* = 41)

Reliability	ICC	95% IC	SEM (elevation)	SEM (%)	MDD (elevation)	MDD (%)
Intra-examiner	0.94	0.89 to 0.97	4.47	6.72	12.40	18.63
Inter-examiner	0.97	0.94 to 0.98	3.07	4.56	8.52	12.64

*ICC* Intraclass correlation coefficient, *CI* Confidence Interval. *SEM* Standard error of measurement, *MDD* Minimum detectable difference

**Table 5:** Correlation between the scores of the 2-min step test (2MST) and the other questionnaires applied (*n* = 41)

Questionnaires	2MST	
	rho	p
Western Ontario and McMaster Universities Osteoarthritis Index		
Pain domain	−0.503	0.001*
Joint stiffness domain	−0.431	0.005*
Physical function domain	−0.536	< 0.001*
Numerical Pain Scale		
Rest	−0.347	0.026*
Movement	−0.478	0.002*
Pain-Related Catastrophizing Thoughts Scale	−0.172	0.281
Pain Self-Efficacy Questionnaire	0.366	0.019*

* Significant correlation (*p* < 0.05, Spearman’s correlation coefficient)

**Fig. 1:**
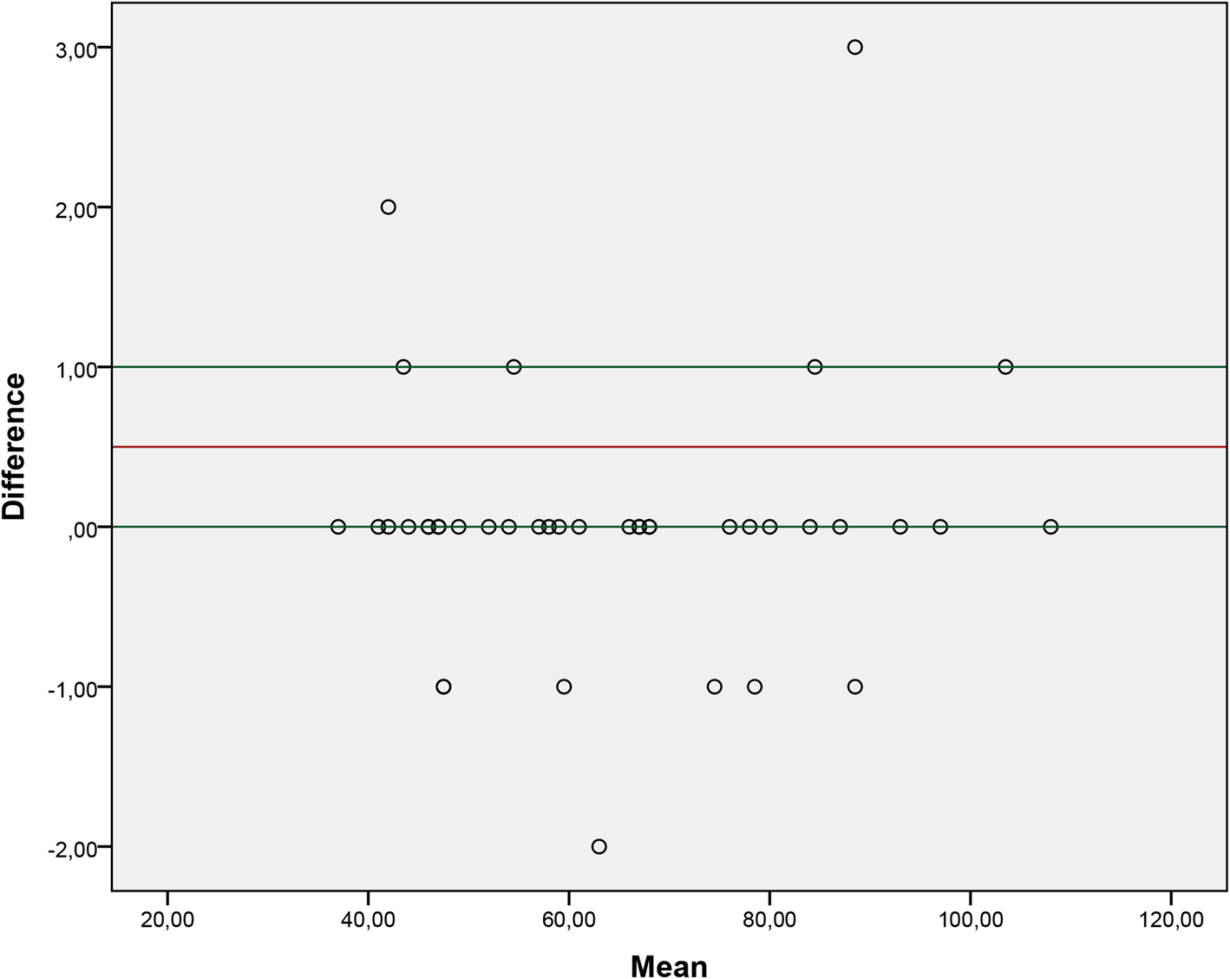
Graph of agreement between the examiner test 1 and the respective analysis through video recording

**Fig. 2:**
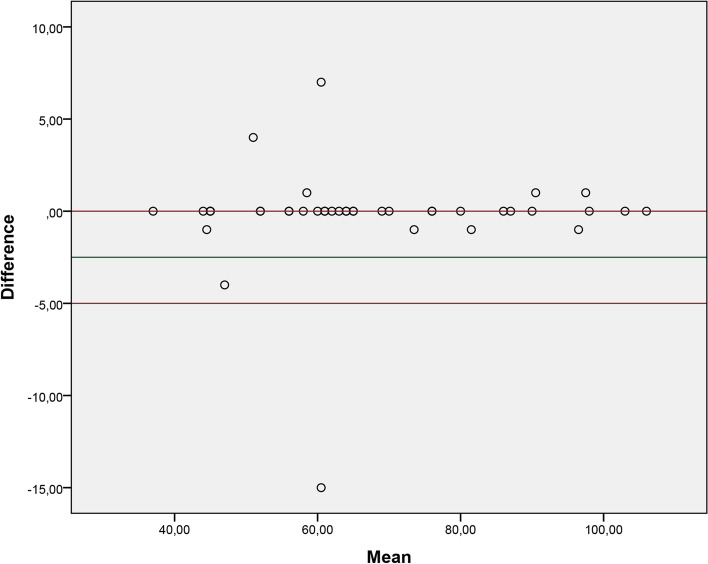
Graph of agreement between the retest of examiner 1 and the respective analysis through video recording

**Fig. 3:**
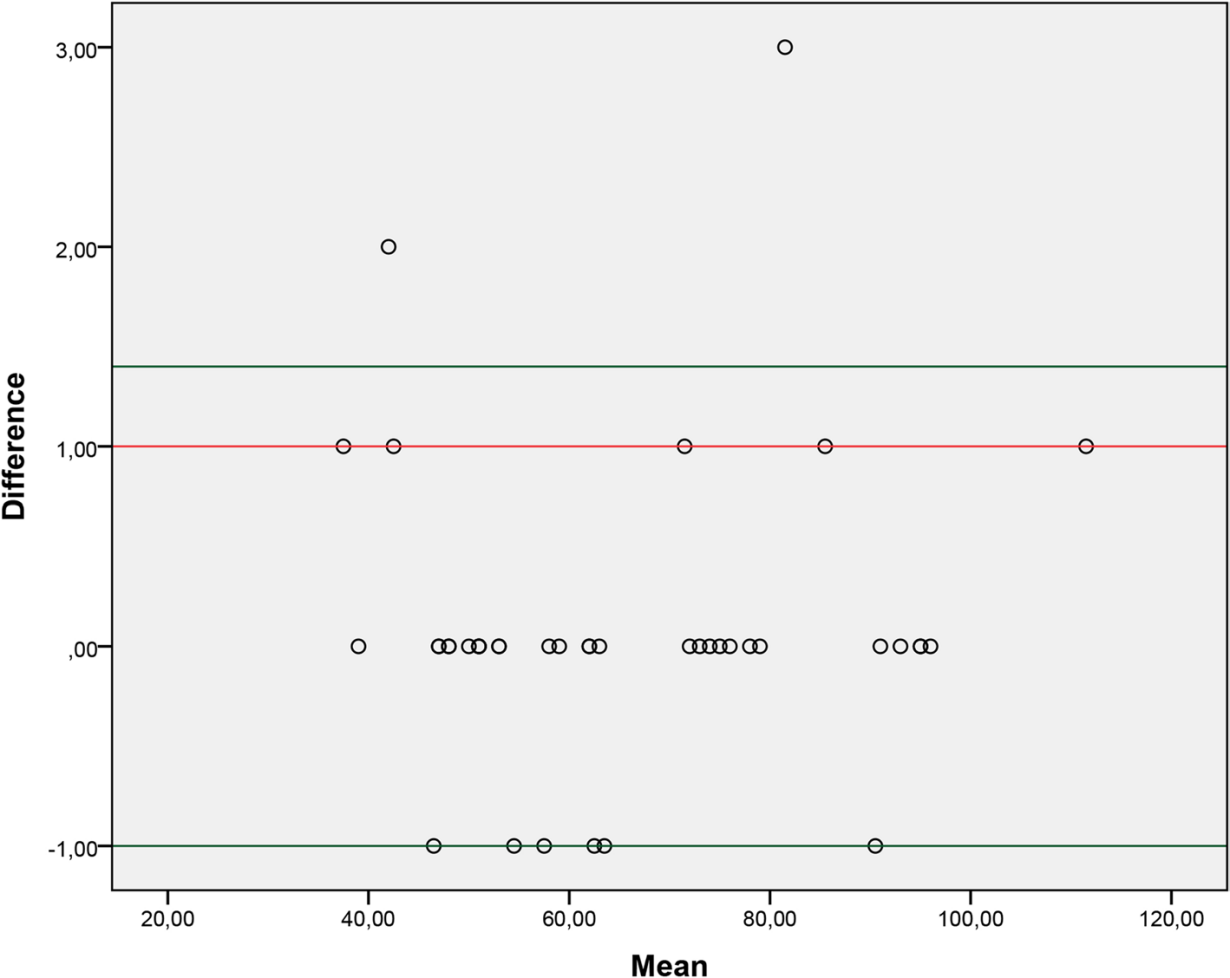
Graph of agreement between the examiner 2 test and the respective analysis through video recording

**Fig. 4:**
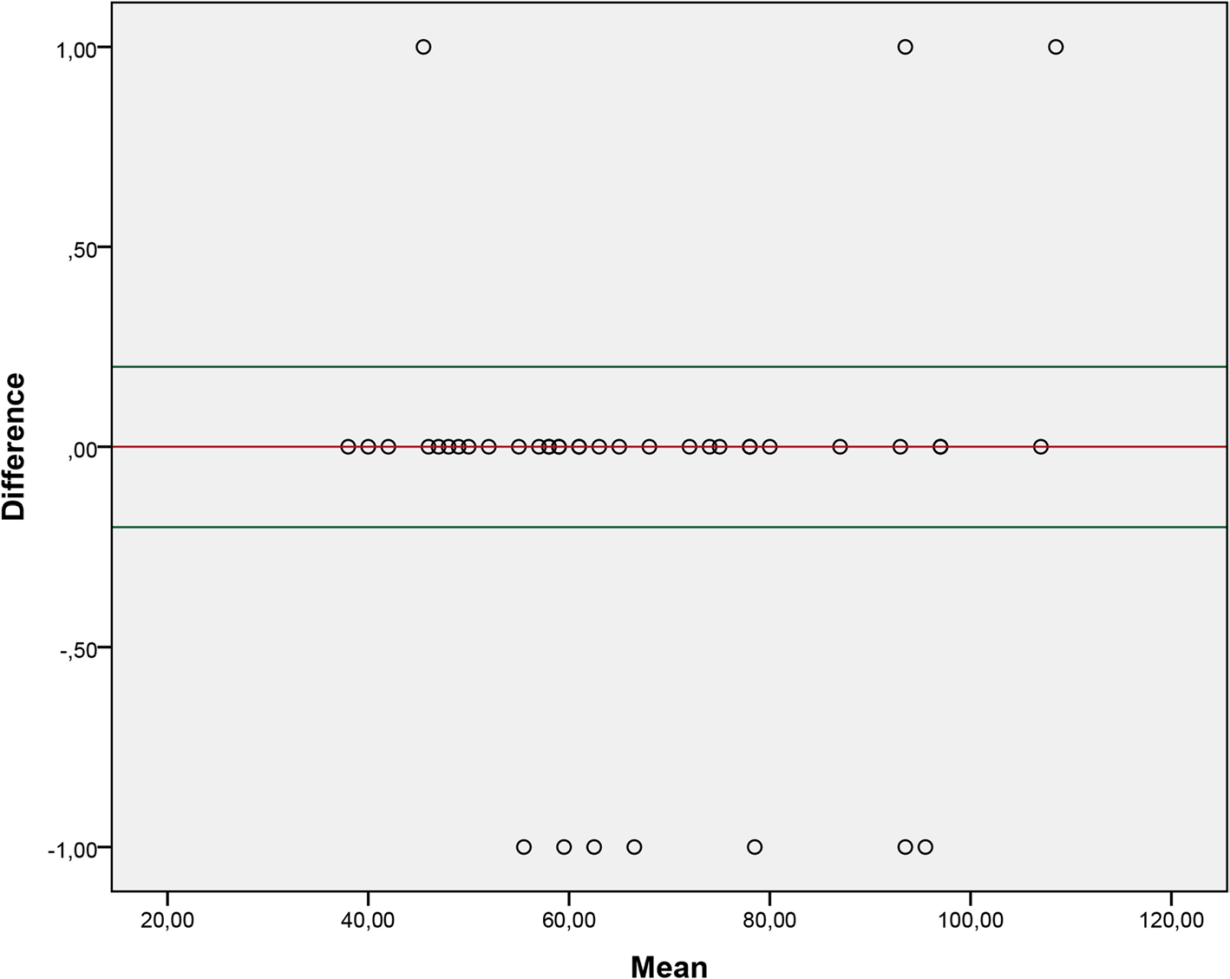
Graph of agreement between the retest of examiner 2 and the respective analysis through video recording

Using the Bland-Altman methodology, Figs. 1 , 2, 3 and 4 show the graphs of acceptable agreement between the assessments made by the examiners and the analyzes performed based on the video recording.

## Discussion

The 2MST showed excellent intra- and inter-examiner reliability in patients with knee OA and was adequately correlated with the NPRS, WOMAC and PSEQ instruments. Other studies have investigated the reliability of 2MST in other populations. The error values inherent to the test were less than 7%. The mean values of the execution in the 2MST were slightly higher in the retest, possibly due to the learning factor; however, the ICC values were adequate.

The scientific literature has only two studies investigating the reliability of the 2MST. Excellent intra-examiner reliability was found in the elderly, with an ICC value of 0.90 [9]. Reliability was considered high in individuals aged between 18 and 44 years, sedentary and active, with an ICC greater than or equal to 0.83 [26]. Our results show higher ICC values than previous studies (ICC ≥ 0.94), probably due to two factors: consistent clinical experience of the examiners (> 10 years) and the completion of training and standardization for 1 month before the start of data collection.

Our study was the first to assess clinimetric properties of 2MST in a population of knee OA. A systematic review conducted by Bohannon and Crouch [27] evaluating the clinimetric properties of 2MST in healthy elderly and elderly people with specific diseases such as heart failure, chronic kidney disease, hypertension, depression and Alzheimer’s disease observed that 2MST was correlated with the level of ability functional, performance on psychocognitive measures, health status and age. However, only one study included in the review addressed the assessment of reliability [9]. Of the 30 articles analyzed, 8 studies showed an increase in repetitions after physical training.

In analyzing the construct validity, we correlated the 2MST score with the NPRS, WOMAC, PCTS and PSEQ scores. With the exception of PCTS, all instruments showed a statistically significant correlation with 2MST, with a magnitude of correlation above 0.30. In addition, a correlation magnitude above 0.50 was found between 2MST and the WOMAC pain and function domains. More than 75% of our hypotheses were confirmed, demonstrating sufficient results for construct validation, according to international guidelines [24]. Therefore, the 2MST measures functionality, with the advantages of being a low cost test, quick execution and not requiring large spaces or specific furniture for its realization.

The relationship between physical function and pain measures has already been investigated in previous studies with patients with knee OA. In a similar way to the present study, Odole et al. [28] identified a correlation magnitude lower than 0.50 in the correlations between physical function and self-efficacy (*r* = 0.35), kinesiophobia (*r* = − 0.43) and catastrophizing (*r* = − 0.28). Investigating catastrophizing in patients with knee OA, Gomes et al. [29] found no correlation with lower limb function, balance and mobility (rho ranging from − 0.22 to 0.25). In addition, other factors are associated with physical-functional performance, such as knee muscle strength, knee flexion range of motion, knee pain, and age [30].

We used self-report instruments (questionnaires and scales) already validated in the population with knee OA to assess the magnitude of correlation with 2MST, and this aspect is an important limitation of our study. Other 2MST validation studies used performance assessment instruments in different populations, showing an adequate correlation with the 1-mile walk time in healthy elderly [10], and weak to moderate correlation with the 6MWT and TUG in a population of hypertensive women [14].

Our study observed adequate agreement between the evaluations carried out in person and the evaluations carried out through video recording. We consider measurement through video as a reference value. The results showed that the mean difference between the two methods is close to 0, which reflects excellent agreement. Therefore, both forms of application of the 2MST can be used, and professionals involved in the rehabilitation of these patients should choose which form of assessment best suits their clinical context.

This study had some limitations that should be mentioned. Our study performed the test and retest keeping the same conditions regarding location, time, fatigue, but we did not control the stability of the clinical symptoms, related to possible pain variation in the 7 to 14-day interval. Reliability analysis by sex was not performed, although we did not observe this analysis in other reliability studies [23, 31, 32]. However, our sample was predominantly female and overweight, and these factors must be considered in the generalization of the results. We did not investigate the influence of the grade in the classification of Kellgren and Lawrence (grade 2 versus 3) on the results of the present study.

## Conclusion

2MST proved to be valid for assessing functional capacity in patients with knee OA, with excellent reliability. The study supports the use of the 2MST in the clinical context and in research with patients with pain, associated with knee OA.

## Data Availability

The data and materials in this paper are available from the corresponding author on request.
